# Copper and zinc solid-solution reinforcement for Ca ions storage

**DOI:** 10.1093/nsr/nwaf108

**Published:** 2025-03-21

**Authors:** Ziqi Sun

**Affiliations:** School of Chemistry and Physics, Queensland University of Technology, Australia

Calcium, a well-known petrogenic element, has significant advantages in terms of cost-effectiveness, especially in comparison to its commercially utilized lithium counterpart (8% vs. 15 ppm in earth crust, respectively) [1]. Currently, lithium-ion batteries (LIBs) dominate the market for sustainable energy production and storage, including consumer electronics and electric vehicles (EVs), playing an indispensable role in our daily lives [2]. However, the sustainability of LIBs is uncertain, considering the uneven global distribution of lithium elements, as well as the unpredictable entanglement of geopolitics. Thus, it is important to investigate next-generation sustainable electrochemical energy-storage technologies, with adequate supply chain stability. Taking calcium ions (Ca^2+^) as charge carriers, the reversible intercalation into hosts could be realized to some extent, but the diffusion kinetics would be far more sluggish than is acceptable, in conventional electrode materials with high theoretical capacities, which greatly weakens the competitiveness of Ca-ion batteries (CIBs) [3].

In a recent proof-of-concept study, scientists used Ca as a metal electrode directly, to couple with advanced catalysts to assemble metal-air battery technologies, and provided proof of the feasibility of Ca-based batteries. Peng and co-workers have developed a calcium-oxygen battery (Ca-O_2_) that can operate at room temperature for 700 charge/discharge cycles [4]. The main point of this work lies in the ionic liquid-based electrolyte used in the system. The highly reversible two-electron redox reaction forms the chemically reactive calcium peroxide (CaO₂) as the discharge product, which can not only achieve a reversible Ca platting process, but also improve the Ca²⁺ kinetics. The researchers also turned the batteries into flexible fibers, which are woven into textile batteries for use in the next generation of wearable systems. Similarly, Sun and co-workers reported a rechargeable Ca/Cl₂ battery based on the reversible redox coupling of CaCl₂ and Cl₂, delivering a high-energy-density rechargeable calcium metal battery system [5]. Specifically, difluoro(oxalato)borate (DFOB⁻) has unique dual functional groups of B−F and C=O, which can promote the dissociation and distribution of Ca²⁺ and chlorine-based species, thus enabling a reversible and rapid redox reaction of CaCl₂/Cl₂. In addition to these strategies, the self-discharge performance and low-temperature performance exhibited by this battery system are expected to break through the bottleneck at the practical-application stage of calcium metal batteries.

**Figure 1.**
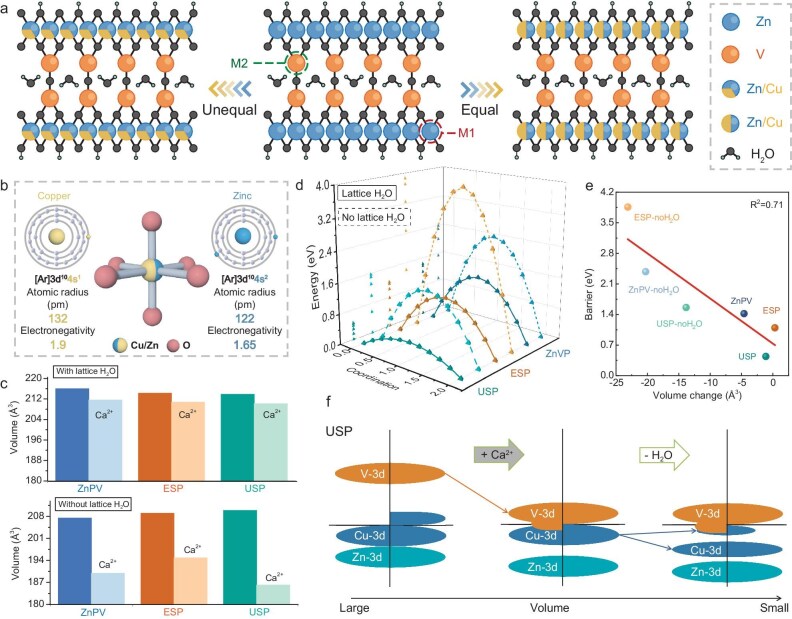
(a) Schematic illustration of solid-solution reconfiguration in different modes: equal and unequal, respectively. (b) Comparison of the physical properties of Cu and Zn elements. (c) Calculation results with regard to the lattice volume of pyrovanadates before and after Ca^2+^ ions intercalation: under the condition of the existence of lattice water in interlayered confinement or lack of lattice water. (d) Energy barriers of pristine pyrovanadates and solid-solution pyrovanadate host. (e) Linear relationship between volume change and diffusion barriers. (f) Mechanism illustration of electronic structure variation with Ca^2+^ ions intercalation and removal of lattice water. Adapted with permission from ref. [6].

The above pioneering studies paid a lot of attention to realizing the favorable and rapid charge carrier (Ca^2+^ and e^−^) transfer from the perspective of electrolyte optimization and conversion-type non-metal species. To further illustrate the practical value of vanadate-based materials, an investigation on host structure design is also worthwhile. In a recent work published in *National Science Review*, Wu and co-workers at Northeast Normal University proposed an allergic reconfiguration strategy to further activate the redox reactivity, in which Cu atoms were introduced into Zinc sites in pyrovanadates to serve as a favorable Ca ions host [6]. The authors selected adjacent Cu and Zn atoms, which belong to the same d-block in the periodic table and share similar properties, including outer-shell electron distribution, atomic radius, and electronegativity. This similarity enables the formation of a robust solid-solution phase, making them an ideal host structure (Fig. 1a and b). Additionally, as a typical layered structure with enriched confinement species, like lattice water and hydroxyls, the solid-solution phase of pyrovanadates shows an advantageous potential for Ca^2+^ storage. By virtue of theoretical calculations, as shown in Fig. 1c, d and e, a linear relationship between the diffusion barrier of Ca^2+^ and the lattice water indicates the importance of the interlayered confined H_2_O molecules in maintaining the crystal structure integrity upon the reversible intercalation of Ca^2+^. Combined with the density of states simulation, the enhanced electronic conductivity for Cu/Zn solid-solution phase host also indicates the effectiveness of this strategy (Fig. 1f).

In summary, Wu and co-workers designed and prepared solid-solution pyrovanadate materials and comprehensively illustrated the solid-solution reinforcement effect, in terms of intrinsic conductivity, interlayered species confinement and electrochemical redox reactivity, for Ca-ion batteries. Through an entropy-driven design, this work offers a promising pathway for advancing multivalent metal-ion battery technologies, particularly in the development of high-performance electrode materials.
